# Comprehensive Biotechnical System for Screening Risk-based Diagnosis of COVID-19 and Post-COVID Syndrome

**DOI:** 10.2478/joeb-2022-0008

**Published:** 2022-09-09

**Authors:** Vladimir Savostyanov, Alexander Kobelev, Ivan Kudashov

**Affiliations:** 1 Faculty of Biomedical Engineering, Bauman Moscow State Technical University, Moscow, Russia

**Keywords:** Bioimpedance, COVID-19, E-health, health care personnel, pathophysiology, post-COVID syndrome, rheography

## Abstract

At present, there are no hardware or biochemical systems that allow to assess the severity of post-COVID syndrome in vivo. The hardware of the proposed biotechnical system is based on routine transthoracic electrical impedance rheography, which makes it possible to register the frequency characteristics of the patient's bioimpedance response to controlled stress stimulation, thereby simultaneously fixing the characteristics of his productive heart, the state of the hemomicrocirculatory bed, the efficiency of the gas transport function of his blood, and also reliably assess personal reactivity and adaptive potential. Subsequent mathematical approximation of the obtained biometric data by an original neural network makes it possible to rank the results obtained and automatically generate a program of medical rehabilitation for a particular patient, depending on the severity of his post-COVID syndrome. The study results proved two reliable physiological signs confirming the presence of latent post-COVID complications: a decrease in the base impedance value for light exercise and an increase in the length of the systolic arc of the rheocardiogram.

## Introduction

Considering the permanent detection of new mutations of the SARS-CoV-2 coronavirus, it is safe to assume that the COVID-19 pandemic will last for several more years (from 3 to 8). And in this situation, it is not the disease itself that becomes dangerous, but its consequences described as “post-COVID syndrome” [1,2].

The long-term presence of a person in post-COVID syndrome due to oxidative stress reactions that trigger the mechanisms of premature apoptosis (death) of cells will ultimately lead to the oncological diseases of organs compromised by COVID-19, with all the ensuing medical, financial and social consequences [3].

Therefore, the development of a special comprehensive biotechnical system (BTS) for assessing the severity of post-COVID syndrome with the calculation of personal rehabilitation since no reliable biochemical markers of post-covid syndrome have been found at present [4,5].

The pathogenesis of the post-COVID syndrome is based on the systemic suffering of the hemomicrocirculatory bed of tissues and organs, triggered by the processes of thrombus formation due to a radical change in the hemorheological properties of blood [6, 7, 8]. And at present, there are no hardware or biochemical systems that make it possible to assess the severity of this process in vivo, only postmortem histological examination [9, 10, 11].

## Research methods and objects

The hardware basis of the proposed complex biotechnical system for screening risk-oriented diagnostics of COVID-19 and the post-COVID syndrome was the method of transthoracic electrical impedance rheography (TEIRG), which allows registering the frequency characteristics of the patient's bioimpedance response to a controlled stressor effect, thereby simultaneously recording the characteristics of his productive work of the heart channel, the effectiveness of the blood gas transport function, as well as to reliably assess its reactivity and adaptive potential.

The method of transthoracic electrical impedance rheography is based on measuring the modulus of electrical impedance of biological tissue $\left|Z\right|=\sqrt{R^{2}+X_{c}^{2}}$when passing a high-frequency (30-150 kHz), low-intensity (0.1 – 10 mA) alternating current, which corresponds to electrical safety standards for the patient [12]. The maximum effective value of the probing current is determined by the electrical safety standard IEC60601 and for a frequency of 100 kHz corresponds to 10 mA [13].

An experimental study of the heart productive work and the state of the hemomicrocirculatory bed by the TEIRG method for diagnosing post-COVID syndrome in volunteers was carried out using the original diagnostic equipment – the ReoCardioMonitor system, which has a Registration Certificate of the Ministry of Health of Russia No. FSR 2010/08720.

The ReoCardioMonitor system has the following technical characteristics:

1. Power supply - 220 V ± 10%, frequency 50 ± 0.5 Hz;
2. Power consumption - no more than 20 W;
3. Electrical safety - class II, B;
4. Rheogram measurement method - tetrapolar;
5. Measuring current - 100 kHz, 8 mA;
6. Measurement range of base impedance - 0 – 240 Ohm;
7. Measurement error of base impedance -± 0.2 Ohm;
8. Measurement range of pulse impedance - ± 0.5 Ohm;
9. Measurement error of pulse impedance - ± 0.1 mOhm;
10. Number of channels - 2 (impedance) + 1 (ECG);
11. ECG lead - from rheographic electrodes.

**Figure 1:**
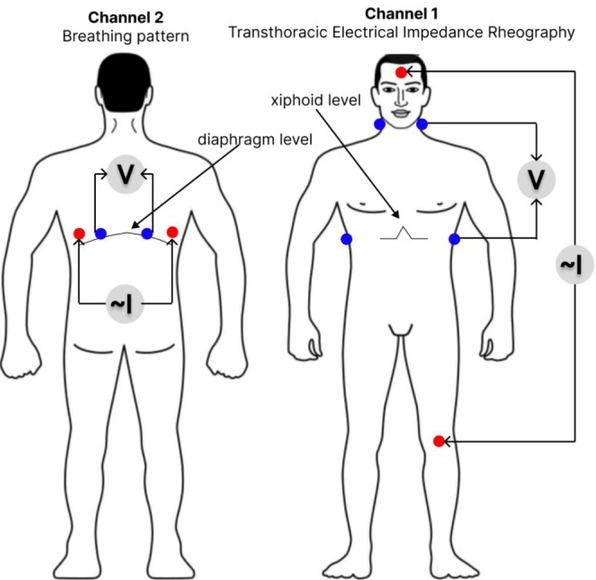
Electrode arrangement diagram.

In technical terms, the “ReoCardioMonitor” system represents a two-channel impedance measuring converter, a patient's cable system, and a personal computer. The first channel (Figure 1, Channel 1) is designed to measure the transthoracic impedance of the chest (base and pulse components, impedance breathing pattern) with an ECG measurement channel from the same impedance electrodes. The transthoracic channel is used to calculate stroke output and cardiac output. The second channel (Figure 1, Channel 2) measures breathing pattern.

The measurement method is tetrapolar. A source that creates an alternating current of high frequency is connected to the current electrodes. The signal is recorded from potential electrodes. With an increase in the distance between the current and potential electrodes, their influence on each other decreases and the accuracy of the measurement result increases [14]. Figure 1 shows the selected scheme of applying the electrodes.

The use of such a scheme for applying electrodes ensures greater uniformity of the current passing through the investigated area and reduces the effect on the measurement result from resistance variations at the electrode-skin interface [15].

Contact with the patient's body was provided with disposable White Sensor 4500 ECG electrodes from the Ambu company, electrode size 50 ✕ 48 mm. The inner electrodes, marked in blue in the diagram, are measuring and registering the voltage change ΔU(t), which is used to calculate and register the impedance signal dZ. External electrodes are required to pass AC ~ I with high frequency and low amplitude. The change in impedance is recorded as a function of time Z(t).

The amount of blood in a body segment is inversely proportional to the electrical impedance of that segment: when the volume of fluid in biological tissues increases, the electrical impedance decreases, when the volume of fluid decreases, the impedance increases. Consequently, the electrical impedance changes according to the content of the fluid volume and changes with each heartbeat [16].

If the blood flow to biological tissues causes a proportional decrease in electrical impedance [17], then the dynamics of the rheographic signal recorded using the RheoCardioMonitor System should contribute to obtaining adequate information about the local and systemic circulation of volunteers who have recovered from COVID-19.

Initially, the study was devoted to the development of a comprehensive biotechnological system (BTS) for determining the state of health and performance of a person against the background of arterial hypertension. The principle of operation of this BTS consists in simultaneous registration of the dynamics of changes in heart rate, respiratory rate and blood pressure by the method of transthoracic electrical impedance rheography (TEIRG) under controlled stress. This methodological approach makes it possible to determine the levels of resistance and reactivity of a biological system, and therefore to assess its adaptive capabilities.

One of the tasks of this work was the validation of the mathematical calculation of blood pressure using data of the rheocardiogram systolic arc.

The research group consisted of 20 volunteers, randomly selected (different sex, weight, height, age). The distribution of volunteers by age, which is a key factor in the development of arterial hypertension, was as follows: 20-29 years old - 1; 30-39 years old - 4; 40-49 years old - 2; 50-59 years old - 2; 60-69 years old - 9; over 70 years old - 2. Moreover, the results of a study of the adaptive capabilities of a person by the method of transthoracic electrical impedance rheography in this research group never showed a drop in the value of the base impedance (BI), provided that for each volunteer, the rheogram was recorded sequentially four times under conditions of controlled physical stress. At the same time, the developed mathematical model, according to the rheogram data, perfectly calculated the values of arterial blood pressure. This was confirmed by the results of dispersion and correlation analysis when compared with blood pressure measurement data using a tonometer. This made it possible to reliably speak of a direct relationship between the rheocardiogram systolic arc length with the arterial blood pressure for adult respondents of any gender, weight, height, and age.

This entire original biotechnical system did an excellent job of its diagnostic functions until repeated tests, which began in the summer of 2021 during the third coronavirus wave caused by the "omicron"-strain. Then some of our volunteers independently turned to us for diagnostic help in determining their adaptive reserves and the degree of performance in order to find out the principles and methods of rehabilitation after they suffered COVID-19.

During the course of repeated tests, the developed mathematical model suddenly began to show some incredibly high values of systolic blood pressure. Moreover, this phenomenon was always recorded immediately after a stressful load and two minutes after recovery. When measured at rest, the arterial blood pressure indicators calculated according to the rheocardiogram systolic arc corresponded to the values measured by the tonometer.

It was during the investigation of this paradox that the drop in the value of the base impedance (BI) at volunteers after a controlled physical stress was first recorded. This phenomenon led us to suggest about the relationship between the change in the base impedance after physical stress and post-COVID syndrome.

Therefore, for carrying out the research project "Comprehensive biotechnical system for screening risk-based diagnosis of COVID-19 and post-COVID syndrome" (Decision of the Ethics Committee of Bauman Moscow State Technical University No. 6 from 2021/10/21) we used a group of 19 volunteers (different sexes , weight and age) who previously participated in our study of an integrated biotechnological system for determining the state of health and performance of a human against the background of arterial hypertension.

All of them were checked up by transthoracic electrical impedance rheography using the “ReoCardioMonitor” system to determine the following physiological parameters: heart rate (HR, bpm), respiratory rate (RR, bpm), rheocardiogram systolic arc length (L, (Ohm·s)^1/2^), the value of the base impedance (BI, Ohm).

**Figure 2:**
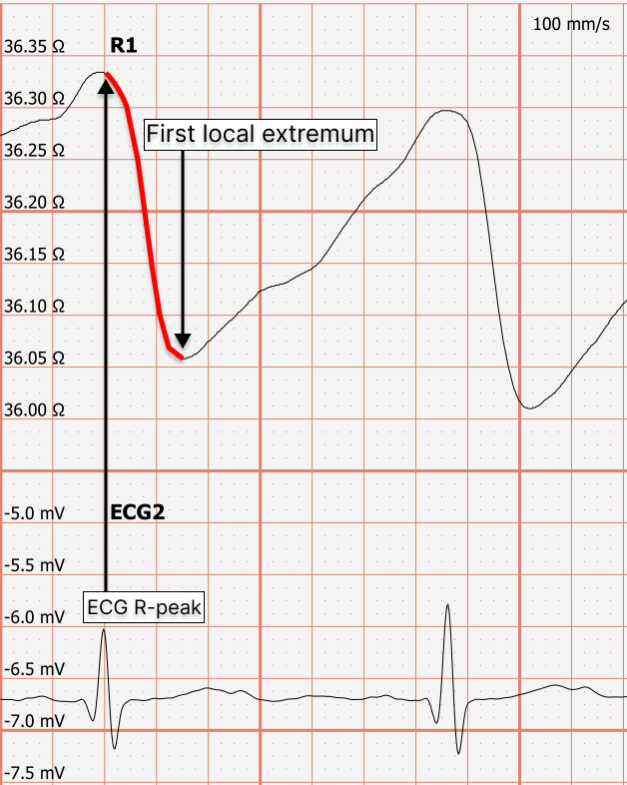
Volunteer participating in the research.

Figure 2 shows the process of systolic arc registration on the transthoracic rheocardiographic examination.

According to the research protocol, the first measurement point was recorded at rest in a sitting position, the second after the maximum breath-holding on inhalation, the third immediately after performing a light physical stress test in the form of squatting or jumping (at the choice of a volunteer) for one minute, and the fourth after two minutes from completing the stress test.

To elucidate the mechanisms of “post-COVID” blood circulation, the obtained results of TEIRG in volunteers of both study groups were approximated by special mathematical algorithms of the diagnostic resource www.healthscreens.guru to obtain a conclusion on:

1. The cardiac output of the left ventricle of the heart (COlv, l / min) and the cardiac output of the right ventricle of the heart (COrv, l / min);
2. The systemic vascular resistance (SVR, dyn·сm·s^-5)^ and the pulmonary vascular resistance (PVR, dyn·сm·s^-5^);
3. The circulating blood volume (CBV, %);
4. Index of cardiovascular adaptation (ICA, units);
5. Index of pulmonary adaptation (IPA, units);
6. Index of endocrine-metabolic burden (IEMB, units);
7. Body mass index (BMI, units);
8. The coefficient of activity of basic metabolism (CABM, units);
9. The coefficient of immediate adaptation (CIA, units);
10. The coefficient of long-term adaptation (CLTA, units);
11. General desadaptative probability (GDP, %).

These integral indicators made it possible to carry out a comprehensive assessment of the functional state of the cardiovascular, respiratory and endocrine-metabolic systems of the volunteers' bodies and their adaptive capabilities.

## Discussion of research results

The average time of one complete checkup (four research points of TEIRG with the parallel determination of blood pressure by an indirect method) was approximately eight minutes.

A sign of randomization for the formation of two study groups “Control” and “Experience” was the dynamics of the response of the base impedance value to a light physical stress test: a decrease in the BI value for the “Experience” study group and an increase (or no changes) in the BI value for the “Control” study group.

As a result, the experience group included 10 volunteers (5 women and 5 men). The average age of the volunteers in the experience group was 65.3 ± 6.1 years, height - 164.0 ± 10.4 cm, weight - 75.9 ± 7.3 kg.

The control group included 9 volunteers (3 women and 6 men). Their average age was 40.1 ± 14.1 years, their height was 177.2 ± 11.0 cm, and their weight was 80.7 ± 11.3 kg.

In the experience group, the drop in the average value of the base impedance was from 43.4 ± 15.03 Ohm to 32.7 ± 12.8 Ohm (u = 397; p <0.01) compared to the base impedance of the control group, where it remained stable or slightly increased: on average from 33.1 ± 5.44 Ohm to 36.4 ± 7.04 Ohm (u = 450.5; p> 0.05).

Following the study protocol, all participants underwent retrospective digital screening diagnostics for COVID-19 using the software www.covid19diagnosis.ru before TEIRG to determine this disease in the history of the volunteer within two months before the start of this study. Subsequently, the received digital diagnosis was compared with the medical documents of the volunteers (discharge letters, PCR tests, CT scan data of the lungs, the presence of specific antibodies to COVID-19).

As a result of the comparison, it turned out that all volunteers of the experience study group (100%) had COVID-19 in their two-month history, while the volunteers of the Control study group did not have this disease in the variant of retrospective digital screening diagnostics, or according to medical data has not been identified. Two volunteers of the control group were identified who, according to the resource www.covid19diagnosis.ru had been ill with COVID-19 about 12 and 18 months ago.

**Figure 3:**
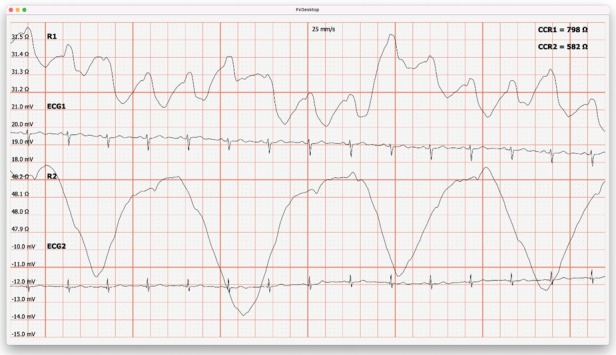
The result of the base impedance before light physical activity is ≈31.3 Ohm in the volunteer of the “Control” group (R1 - base impedance, ECG - ECG signal, R2 - breathing pattern).

**Figure 4:**
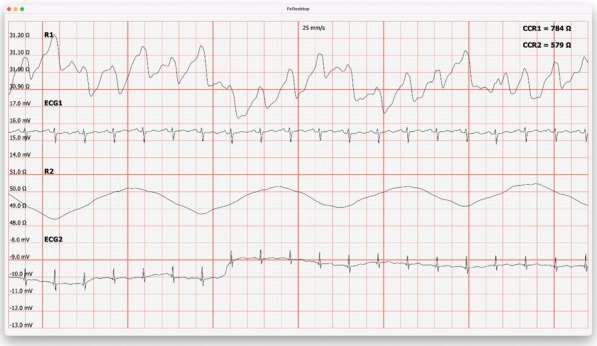
The result of the base impedance after light physical activity is ≈31.0 Ohm in the volunteer of the “Control” group (R1 - base impedance, ECG - ECG signal, R2 - breathing pattern).

**Figure 5:**
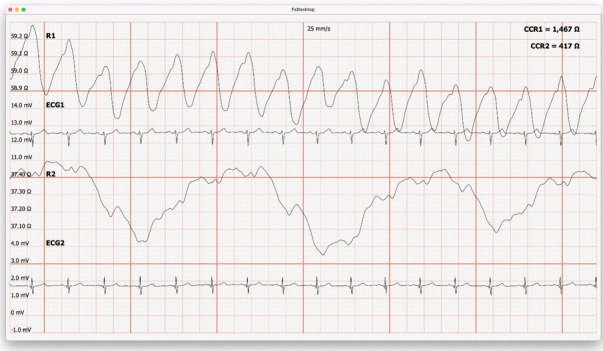
The result of the base impedance before light exercise is ≈58.9 Ohm for the volunteer of the “Experience” group (R1 - base impedance, ECG - ECG signal, R2 - breathing pattern).

**Figure 6:**
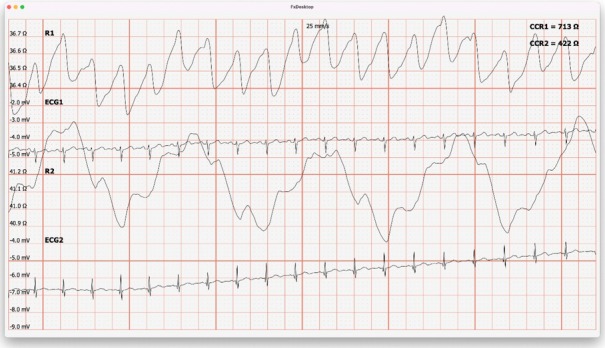
The result of the base impedance after light physical exertion is ≈36.5 Ohm in the “Experience” group volunteer (R1 - base impedance, ECG - ECG signal, R2 - breathing pattern).

Figures 3, 4 and 5, 6 show that the first conclusion from the results of this study was the understanding that in the early period after COVID-19, patients showed a significant decrease in baseline impedance during light exercise.

The reason for this is most likely the massive suffering of the patency of the capillaries of the hemomicrocirculatory bed (HMCB) due to micro thrombosis caused by the new coronavirus infection SARS-CoV-2, which is confirmed by numerous pathological studies [18,19]. Fluid retention at the HMCB level leads to tissue hypoxia, which triggers oxidative stress reactions with the accumulation of free radicals that damage biomembranes, and thus initiate cell apoptosis [20]. The prolonged existence of biological tissues in this state ultimately leads to their fibrosis, followed by sclerosis and persistent suffering of their blood supply, which ultimately manifests itself in the irreversible loss of function of specific anatomical elements (joints, ligaments, intervertebral discs), individual organs (lungs, heart, liver, kidney, pancreas, brain) and even entire organ systems (cardiovascular, respiratory, endocrine-metabolic, central nervous system), which radically affects the quality of life of a patient who has recovered from COVID-19 [21]. And the main task in the medical rehabilitation of such patients is to understand what is leading: the exacerbation of chronic diseases already existing in the anamnesis or the primary suffering of hemomicrocirculation caused by SARS-CoV-2 [22,23]. Because the tactics of medical rehabilitation of a patient in this situation will be radically different [24].

Considering the previously described randomization procedure, two representative data sets were obtained to perform a statistical comparison of the physiological parameters of volunteers who did not have COVID-19 (control - 36 full-fledged mathematical lines), and volunteers who had had COVID-19 (experience - 38 full-fledged mathematical lines).

The numerical data of these statistical samples were found to be sufficient and representative.

To perform statistical analysis, nonparametric tests were used: for variance comparison - the Mann-Whitney test (u); for the correlation one - the calculation of the Spearman's rank correlation coefficient (rs).

The results of comparing the average values of indicators of central hemodynamics, rheocardiogram and integral indicators of the functional state of the cardiovascular, respiratory and endocrine-metabolic systems of the body of the volunteers of the control and experimental groups with their adaptive capabilities are presented in Table 1 and Table 2.

**Table 1:** Integral Indicators of The Functional State of The Cardiovascular and Respiratory Systems.

Group	COLV, l/min	CORV, l/min	SVR, dyn·сm·s^-5^	PVR, dyn·сm·s^-5^	CBV, %	ICA, units	IPA, units
“Control”	6,4 ± 0,58	5,5 ± 1,05	993 ± 269	948 ± 293	97,6 ± 11,4	0,63 ± 0,15	1,0 ± 0,57
“Experience”	6,7 ± 0,6	6,1 ± 1,3	1004 ± 204	973 ± 211	103,5 ± 11,8	0,59 ± 0,11	0,97 ± 0,34
Statistical indicator	u = 558	u = 511	u = 602	u = 559	u = 504	u = 633	u = 654
Error	p > 0,05	p < 0,05	p > 0,05	p > 0,05	p < 0,05	p > 0,05	p > 0,05

**Table 2:** Integral Indicators of The Functional State of The Endocrine-Metabolic System and Adaptive Capabilities.

Group	BMI, units	IEMB, units	CABM, units	CIA, units	CLTA, units	GDP, %
“Control”	25,9 ± 4,5	1,68 ± 0,72	1,35 ± 0,27	1,72 ± 0,8	0,99 ± 0,27	50,5 ± 21,8
“Experience”	28,2 ± 2,2	1,75 ± 0,35	1,49 ± 0,18	1,79 ± 0,6	0,92 ± 0,18	57,2 ± 14,8
Statistical indicator	u = 479	u = 495	u = 444	u = 580	u = 616	u = 603
Error	p < 0,01	p < 0,05	p < 0,01	p > 0,05	p > 0,05	p > 0,05

So, the first statistically significant difference in the performance of the experimental and control groups recorded in the study was a significant drop in the base impedance in volunteers with COVID-19 against the background of their physical stress test. This condition can be characterized as a sudden accumulation of fluid at the levels of the hemomicrocirculatory bed (HMCB), caused by physical activity, which led to an increase in the electrical conductivity of biological tissues.

Under normal conditions, HMCB capillaries’ function “step-by-step” 25-30% of their total number. In case of a stressful need, new “portions” of capillaries are connected to active tissue gas exchange, which contributes to the successful implementation of the increasing tension of the gas transport function of the circulatory system. After recovering from stress, the number of actively working capillaries decreases to normal [25].

With COVID-19, the number of normally functioning capillaries progressively decreases depending on the severity of the disease, causing inhibition of tissue gas exchange and increased suffering in the gas transport function of blood. Considering the increased tropism of the SARS-CoV-2 coronavirus to vascular endothelial cells and its direct toxic effect on the red hematopoietic germ, it was surprising that there were no statistically significant differences in the total peripheral vascular resistance in both circles of blood circulation in the volunteers of the experimental and control research groups. However, an increase in the minute volume in the right ventricle of the heart and the volume of circulating blood in volunteers who had recovered from COVID-19 (Table I) confirmed a hypervolemic reaction of blood circulation with a tendency to develop pulmonary hypertension in them with this disease. Nevertheless, in addition to the weighted endocrine-metabolic background (Table 2), there were no other pathological abnormalities in the cardiovascular and respiratory systems in the volunteers of the experimental research group compared with the volunteers in the control research group.

This means that there are currently no specific clinical symptoms or functional signs for diagnosing the severity of the condition of patients after COVID-19 at the primary health care level [26]. And the patients themselves after COVID-19 in the early rehabilitation period after the disease, usually do not present active complaints [27].

This medical fact was confirmed by the absence of statistically significant differences in the mean values of the coefficient of immediate adaptation (CIA) (1.79 ± 0.6 units versus 1.72 ± 0.8 units; u = 580; p> 0.05), the coefficient longterm adaptation (CLTA) (0.92 ± 0.18 units versus 0.99 ± 0.27 units; u = 616; p > 0.05) and general adaptation probability (GAP) (57.2 ± 14, 8% versus 50.5 ± 21.8%; u = 603; p > 0.05) in the volunteers of the “Experience” and “Control” study groups (Table 2). At the same time, none of the volunteers of the “Experience” group actively complained about their condition.

A varied clinical performance, called the “post-COVID syndrome”, manifests itself in patients 2-3 months after COVID-19, when it is no longer possible to establish a causal relationship with this disease. It is for this reason that the pathogenesis and specific clinical signs of the post-COVID syndrome have not yet been differentiated [28].

This study did not find statistically significant differences in systolic (SBP) and diastolic (DABP) arterial blood pressure, heart rate (HR) and respiratory rate (RR) between control and COVID-19 volunteers (Table 3)".

**Table 3:** Indicators of Central Hemodynamics, Respiration and Rheocardiogram Data.

Group	SABP, mm hg	DABP, mm hg	HR, bpm	RR, bpm	L_0_, (Ohm·s)^1/2^
“Control”	141,4 ± 19,3	81,7 ± 9,8	83,6 ± 23,2	18,6 ± 4,1	0,329 ± 0,07
“Experience”	137,6 ± 22,4	79,5 ± 9,1	83,0 ± 15,4	17,2 ± 3,0	0,386 ± 0,12
Statistical indicator	u = 598	u = 588	u = 612	u = 577	u = 492
Error	p > 0,05	p > 0,05	p > 0,05	p > 0,05	p<0,05

Thus, based on the results of this study, in addition to a significant drop in the baseline impedance to physical activity due to fluid retention at the HMCB level due to capillary microthrombosis, statistically significant differences were also found in the length of the systolic arc of the rheocardiogram in volunteers with COVID-19 (0.386 ± 0.12 Ohm versus 0.329 ± 0.07 Ohm; u = 492; p < 0.05), and there was also a significant increase in the cardiac output of the right ventricle of heart (6.1 ± 1.3 L / min versus 5.5 ± 1.05 l / min; u = 511; p < 0.05) and circulating blood volume (103.5 ± 11.8% versus 97.6 ± 11.4%; u = 504; p < 0.05) against the background of increased body mass index (BMI), index of endocrine-metabolic burden (IEMB) and coefficient of activity of basic metabolism (CABM).

To study these clinical phenomena, Spearman's rank correlation analysis (rs) was used.

Based on its results, it became clear that in the “Control” study group, the heart rate (HR) and the length of the systolic arc of the rheocardiogram (L_0_) are strongly inversely correlated (rs = -0.347; p <0.05). The respiratory rate (RR) are strongly inversely correlated with L_0_ (rs = -0.55; p <0.01).

The very same heart rate is associated with a reliable strong direct correlation with RR (rs = 0.534; p <0.01).

And this is a normal in vivo picture, when the effective gas exchange function of blood circulation at the level of HMCB (L_0_) is maintained at the expense of heart rate and RR, regulated by the vasculomotor and respiratory centres of the medulla oblongata.

The medulla oblongata is important in the regulation of respiration, cardiac activity, the state of blood vessels, and sweating. It is in the medulla oblongata that the centres of all these functions are localized. A feature of some of them (respiratory and vasculomotor) is that they are excited both reflexively by nerve impulses coming from the periphery, and by chemical stimuli acting directly on them [29].

In the “Experience” study group, a radically different relationship between clinical signs was revealed.

The length of the systolic arc of the rheocardiogram (L_0_) was strongly correlated inversely with the systolic component of blood pressure (SBP) (rs = -0.424; p <0.01) and heart rate (HR) (rs = -0.614; p <0.01). At the same time, heart rate was found to be associated with SBP (rs = 0.612; p <0.01), but not with the respiratory rate (RR) (rs = -0.126; p> 0.05), which completely contradicts the fundamental basics of blood circulatory physiology.

It turns out that in patients who have undergone COVID-19, the regulatory function of the vasculomotor and respiratory centers of the medulla oblongata is paradoxically disrupted, as a result of which the clinical indicators of central hemodynamics (blood pressure, heart rate, respiratory rate) remain in the normal range, creating the illusion of somatic well-being, while the biological tissues damaged by SARS-CoV-2 undergo fibrous involution with subsequent sclerotic degradation due to the previously transferred “cytokine storm” and increasing chronic tissue hypoxia caused by blocking the mechanisms of gas exchange and blood circulation at the levels of the hemomicrocirculatory bed due to microthrombosis.

## Conclusions

The study results proved that people who have undergone COVID-19 have two reliable physiological signs confirming the presence of latent post-COVID complications: a decrease in the base impedance (BI) value for light exercise and an increase in the length of the systolic arc of the rheocardiogram (L_0_), which allows the use of the method of transthoracic electrical impedance rheography (TEIRG) for the preventive diagnosis of the development of “post-COVID syndrome”.
